# The rs2147578 C > G polymorphism in the *Inc-LAMC2–1:1* gene is associated with increased neuroblastoma risk in the Henan children

**DOI:** 10.1186/s12885-018-4847-y

**Published:** 2018-10-03

**Authors:** Tianyou Yang, Zhuorong Zhang, Jiao Zhang, Tianbao Tan, Jiliang Yang, Jing Pan, Chao Hu, Jiahao Li, Huimin Xia, Jing He, Yan Zou

**Affiliations:** 1 Department of Pediatric Surgery, Guangzhou Women and Children’s Medical Center, Guangzhou Medical University, 9 Jinsui Road, Guangzhou, 510623 Guangdong China; 2 Department of Pediatric Surgery, The First Affiliated Hospital of Zhengzhou University, Zhengzhou, 450052 Henan China

**Keywords:** rs2147578, Neuroblastoma, Long non-coding RNA, Polymorphism

## Abstract

**Background:**

The rs2147578 C > G polymorphism in the long non-coding RNA gene *Lnc-LAMC2–1:1* is associated with increased susceptibility to a few types of cancers. However, its role in neuroblastoma has not been evaluated yet.

**Methods:**

We investigated the association between the *lnc-LAMC2–1:1* rs2147578 C > G polymorphism and neuroblastoma susceptibility in Chinese Han populations. A total of 393 neuroblastoma cases and 812 healthy individuals from the Henan and Guangdong provinces were enrolled and subjected to genotyping. Odds ratio (OR) and 95% confidence interval (CI) were used to determine the strength of the association of interest.

**Results:**

Combined analysis revealed that the *lnc-LAMC2–1:1* rs2147578 C > G polymorphism was associated with increased neuroblastoma susceptibility (CG vs. CC: adjusted OR = 1.33, 95% CI = 1.01–1.75, *P* = 0.045; CG/GG vs. CC: adjusted OR = 1.34, 95% CI = 1.03–1.74, *P* = 0.028). In stratification analysis, children under 18 months with rs2147578 CG/GG genotypes had an increased neuroblastoma risk (adjusted OR = 1.70, 95% CI = 1.08–2.67, *P* = 0.022). Females with rs2147578 CG/GG genotypes also had increased neuroblastoma susceptibility (adjusted OR = 2.08, 95% CI = 1.37–3.18, *P* = 0.0007). In addition, children with *lnc-LAMC2–1:1* rs2147578 CG/GG genotypes were prone to develop earlier stages of neuroblastoma (adjusted OR = 1.46, 95% CI = 1.01–2.12, *P* = 0.046).

**Conclusions:**

The *Lnc-LAMC2–1:1* rs2147578 C > G polymorphism may contribute to increased neuroblastoma susceptibility in children of Henan province.

**Electronic supplementary material:**

The online version of this article (10.1186/s12885-018-4847-y) contains supplementary material, which is available to authorized users.

## Background

Neuroblastoma is the most common malignant extracranial solid tumor in children, accounting for 7–10% of all tumors [1, 2]. Neuroblastoma originates from neural crest precursor cells of the sympathetic nervous system and is mainly located in the adrenal medulla, paraspinal ganglia, and sympathetic trunk [3–5]. The outcome of neuroblastoma is affected by several factors such as age of onset, pathological subtype, International Neuroblastoma Staging System (INSS) stage, N-myc status, DNA ploidy, and structural chromosomal aberrations [3–5].

Genetic factors are critically important in neuroblastoma tumorigenesis. Approximately 1% of the patients have a family history of neuroblastoma, and are carriers of certain genetic mutations. For instance, *anaplastic lymphoma kinase* (*ALK*) and *PHOX2B* gene variants are among the predisposing factors to familial neuroblastoma [6–8]. Evidence of genome-wide association studies (GWASs) of sporadic cases also suggests that genetic factors may be involved in the pathogenesis of neuroblastoma [9, 10]. These studies indicate an important role of genetic characteristics in the tumorigenesis of this disease.

Long non-coding RNAs (lncRNAs) are mRNA-like molecules whose genes belong to the non-protein coding genome. LncRNAs are involved in many biological processes such as gene imprinting, epigenetic regulation, translational regulation, splicing, and aging [11–15]. LncRNAs are also involved in apoptosis and cell differentiation, which are critical in tumorigenesis [16, 17]. LncRNAs such as *HULC*, *PCAT-1*, *HOTAIR*, *ANRIL*, and *H19* are found to play important roles in cancer development [17]. Single nucleotide polymorphism (SNP) at *HULC* is associated with decreased hepatocellular carcinoma risk [18]. GWAS show that neuroblastoma patients with the G allele of SNP rs6939340, which is located in the lncRNA LOC729177 gene, have a high risk of metastasis and poor outcome [19, 20]. These studies indicate that evaluation of lncRNA gene polymorphism would be of great value in the risk assessment of neuroblastoma.

The rs2147578 C > G polymorphism in the lncRNA gene *Lnc-LAMC2–1:1* is associated with susceptibility to several types of cancer, and functional polymorphism in *lnc-LAMC2–1:1* may confer a high risk of colorectal cancer through affecting miRNA binding [21]. Moreover, the *lnc-LAMC2–1:1* rs2147578 polymorphism is also considered a possible risk factor for acute lymphoblastic leukemia (ALL) in children [22]. However, few studies have focused on this polymorphism in neuroblastoma. Here we hypothesized that the *lnc-LAMC2–1:1* rs2147578 C > G polymorphism may contribute to neuroblastoma susceptibility, and we tested our hypothesis via a case-control study.

## Materials and methods

### Study subjects

The subjects enrolled were described in previous studies [23, 24]. Briefly, 393 neuroblastoma patients and 812 cancer-free controls were enrolled from two different provinces of China: 275 neuroblastoma patients and 531 controls from the Guangzhou Women and Children’s Medical Center in Southern China, and 118 neuroblastoma patients and 281 controls from the Henan province in Northern China [25, 26]. Diagnosis and clinical stages of neuroblastoma were assigned according to the Shimada system and the international criteria for neuroblastoma staging system [27, 28]. Healthy controls had no history of malignancies and were matched to the neuroblastoma cases in terms of age (±5 years), gender, ethnicity, and geographical region. Both cases and controls were unrelated Chinese Han individuals living in the Guangdong and Henan provinces of China.

This study was approved by the Institutional Review Board of Guangzhou Women and Children’s Medical Center (Guangzhou, China), and written informed consent was obtained from the parents of the participants or their legal guardians for the use of their children’s medical data and biological samples.

### SNP selection and genotyping

The *Lnc-LAMC2–1:1* rs2147578 C > G polymorphism was genotyped using the TaqMan real-time PCR system on a 7900 Sequence Detection System (Applied Biosystems, Foster City, CA), as described previously [29–31]. Briefly, high-quality DNA samples were genotyped using Taqman real-time PCR method on a 7900 HT sequence detector system. The call rate for the SNPs was 99%, which met the pre-set criterion. For quality control, eight duplicate positive and eight negative controls without DNA were used in each 384-well plate [32, 33]. Additionally, 10% samples were randomly selected and repeated, and the reproducibility was 100% concordant.

### Statistical analysis

All statistical tests were two-sided, with a significance level of *P* < 0.05. All statistical analyses were performed using SAS software (Version 9.4; SAS Institute, Cary, NC, USA). Two-sided *χ*^*2*^ tests were used to analyze demographic data and genotype frequencies. The Hardy-Weinberg equilibrium was assessed using the goodness-of-*χ*^*2*^ test. Odds ratios (OR) and 95% confidence intervals (CIs) were calculated using the Woolf approximation method to evaluate association between the *lnc-LAMC2–1:1* rs2147578 C > G polymorphism and neuroblastoma susceptibility. Crude and age- and gender-adjusted OR were evaluated using the unconditional logistic regression method.

## Results

### Demographic characteristics of the study population

A total of 275 cases of neuroblastoma and 531 health controls in Guangdong province and 118 neuroblastoma cases and 281 health controls in Henan province were evaluated (Additional File 1 Table S1). Age and gender distributions were similar between cases and controls in both Guangdong and Henan province subgroups (*P* > 0.05). Distribution of clinical stages and sites of tumor origin are also listed in Additional file 1 Table S1.

### *Lnc-LAMC2–1:1* rs2147578 C > G polymorphism and susceptibility to neuroblastoma

Genotype and allele frequencies of the *lnc-LAMC2–1:1* rs2147578 C > G polymorphism and associations with neuroblastoma risk are summarized in Table 1. In both combined and subgroup analyses, the genotype distribution of the *lnc-LAMC2–1:1* rs2147578 C > G polymorphism in the controls and cases were consistent with Hardy-Weinberg equilibrium (*P* = 0.164 for combined subjects, *P* = 0.279 for Guangdong province, and *P* = 0.386 for Henan province).

**Table 1:** Genotype distribution of the *lnc-LAMC2–1:1* rs2147578 C > G polymorphism and neuroblastoma susceptibility

Genotype	Case	Control	*P* ^a^	Crude OR (95% CI)	*P*	Adjusted OR (95% CI) ^b^	*P* ^b^
Guangdong province (HWE = 0.279)							
CC	88 (32.00)	195 (36.79)		1.00		1.00	
CG	130 (47.27)	243 (45.85)		1.19 (0.85–1.65)	0.312	1.18 (0.85–1.64)	0.332
GG	57 (20.73)	92 (17.36)		1.37 (0.91–2.08)	0.135	1.38 (0.91–2.10)	0.127
Additive			0.305	1.17 (0.96–1.44)	0.124	1.18 (0.96–1.44)	0.120
Dominant	187 (68.00)	335 (63.21)	0.177	1.24 (0.91–1.68)	0.177	1.23 (0.91–1.68)	0.183
Recessive	218 (79.27)	438 (82.64)	0.243	1.25 (0.86–1.80)	0.244	1.26 (0.87–1.82)	0.223
Henan province (HWE = 0.386)							
CC	29 (24.58)	99 (35.36)		1.00		1.00	
CG	67 (56.78)	129 (46.07)		**1.77 (1.07–2.95)**	**0.027**	**1.73 (1.03–2.89)**	**0.037**
GG	22 (18.64)	52 (18.57)		1.44 (0.76–2.76)	0.266	1.42 (0.74–2.74)	0.291
Additive			0.085	1.25 (0.92–1.70)	0.158	1.23 (0.90–1.69)	0.186
Dominant	89 (75.42)	181 (64.64)	0.036	**1.68 (1.03–2.73)**	**0.037**	**1.64 (1.004–2.68)**	**0.048**
Recessive	96 (81.36)	228 (81.43)	0.986	1.01 (0.58–1.75)	0.986	1.00 (0.57–1.74)	1.000
Combined (HWE = 0.164)							
CC	117 (29.77)	294 (36.30)		1.00		1.00	
CG	197 (50.13)	372 (45.93)		**1.33 (1.01–1.75)**	**0.042**	**1.33 (1.01–1.75)**	**0.045**
GG	79 (20.10)	144 (17.78)		1.38 (0.97–1.95)	0.071	1.38 (0.97–1.95)	0.074
Additive			0.080	**1.19 (1.01–1.41)**	**0.043**	**1.19 (1.004–1.41)**	**0.045**
Dominant	276 (70.23)	516 (63.70)	0.025	**1.34 (1.04–1.74)**	**0.025**	**1.34 (1.03–1.74)**	**0.028**
Recessive	314 (79.90)	666 (82.22)	0.331	1.16 (0.86–1.58)	0.331	1.16 (0.86–1.58)	0.333

^a^ *χ*^*2*^ test for genotype distribution in neuroblastoma cases and cancer-free controls

^b^ Adjusted for age and gender

*OR* odds ratio, *CI* confidence interval

The values were in bold if the 95% CI excluded 1, or *P*<0.05

No significant difference in CC, CG, and GG genotype distributions was found in the Guangdong subgroup, indicating that the *Lnc-LAMC2–1:1* rs2147578 C > G polymorphism is not associated with neuroblastoma risk in the Guangdong study population. However, the *lnc-LAMC2–1:1* rs2147578 C > G polymorphism was associated with increased neuroblastoma risk in the Henan population (CG vs. CC: adjusted OR = 1.73, 95% CI = 1.03–2.89, *P* = 0.048; CG/GG vs. CC: adjusted OR = 1.64, 95% CI = 1.004–2.68, *P* = 0.048).

The combined analysis showed that the distribution of the CG genotype was significantly higher in the neuroblastoma group (adjusted OR = 1.33, 95% CI = 1.01–1.75, *P* = 0.045), indicating that the *lnc-LAMC2–1:1* rs2147578 CG/GG genotype carriers had an increased risk of neuroblastoma (CG/GG vs. CC: adjusted OR = 1.34, 95% CI = 1.03–1.74, *P* = 0.028).

### Stratification analysis of the *lnc-LAMC2–1:1* rs2147578 C > G polymorphism and neuroblastoma risk

Stratification analyses according to age, gender, site of origin, and clinical stage were further conducted. The CG/GG genotypes in children younger than 18 months were associated with increased neuroblastoma risk (adjusted OR = 1.70, 95% CI = 1.08–2.67, *P* = 0.022). Females with the CG/GG genotypes were associated with increased neuroblastoma risk (adjusted OR = 2.08, 95% CI = 1.37–3.18, *P* = 0.0007). In addition, Individuals with the CG/GG genotypes tended to be in an earlier clinical stage of neuroblastoma (adjusted OR = 1.46, 95% CI = 1.01–2.12, *P* = 0.046). Finally, no significant association between the *lnc-LAMC2–1:1* rs2147578 C > G polymorphism and the site of tumor origin was found (Table 2).

**Table 2:** Stratification analysis of the association between the *lnc-LAMC2–1:1* rs2147578 C > G polymorphism and neuroblastoma susceptibility for combined subjects

Variable	rs2147578 (case/control)		Crude OR	*P*	Adjusted OR ^a^	*P* ^a^
	CC	CG/GG	(95% CI)		(95% CI)	
Age, month						
≤ 18	35/120	91/184	**1.70 (1.08–2.67)**	**0.022**	**1.70 (1.08–2.67)**	**0.022**
> 18	82/174	185/332	1.18 (0.86–1.63)	0.302	1.18 (0.86–1.62)	0.312
Gender						
Female	39/130	129/211	**2.04 (1.34–3.10)**	**0.0009**	**2.08 (1.37–3.18)**	**0.0007**
Male	78/164	147/305	1.01 (0.73–1.42)	0.938	1.00 (0.72–1.40)	0.981
Site of origin						
Adrenal gland	43/294	110/516	1.46 (1.00–2.13)	0.052	1.41 (0.96–2.07)	0.077
Retroperitoneal region	31/294	56/516	1.03 (0.65–1.63)	0.903	1.04 (0.66–1.66)	0.863
Mediastinum	32/294	77/516	1.37 (0.89–2.12)	0.157	1.39 (0.90–2.16)	0.138
Others	9/294	27/516	1.71 (0.79–3.68)	0.171	1.75 (0.81–3.78)	0.155
Clinical stage						
I + II + 4 s	46/294	116/516	1.44 (0.99–2.08)	0.055	**1.46 (1.01–2.12)**	**0.046**
III + IV	68/294	143/516	1.20 (0.87–1.65)	0.272	1.16 (0.84–1.61)	0.362

^a^ Adjusted for age and gender

*OR* odds ratio; *CI* confidence interval

The values were in bold if the 95% CI excluded 1, or *P*<0.05

## Discussion

In this study, we investigated the association between the *lnc-LAMC2–1:1* rs2147578 C > G polymorphism and neuroblastoma susceptibility in Chinese Han populations. We found that the *lnc-LAMC2–1:1* rs2147578 C > G polymorphism is associated with increased neuroblastoma susceptibility. Specifically, females and children younger than 18 months with specific genotypes in the *lnc-LAMC2–1:1* rs2147578 C > G polymorphism are at an increased risk of neuroblastoma. Fortunately, individuals with the CG/GG variation tended to have the earlier stages of neuroblastoma.

Neuroblastoma accounts for approximately 15% of all childhood cancer mortality, and understanding the underlying mechanisms of this disease would be of great value for diagnosis and treatment [1, 2]. Genetic variants are critical in neuroblastoma tumorigenesis and disease progression. Missense mutations in *PHOX2B*, located on chromosome 4p, were the first germline mutations identified to be associated with neuroblastoma predisposition. Other genetic mutations, such as SNPs in the *ALK* and *BARD1* genes and copy number polymorphism of *NBPF23*, may also have a role in neuroblastoma development [6–8].

LncRNAs are involved in many biological processes, with *LncRNA-MALAT1* and *GAS5* being reported to mediate cell invasion, migration, and apoptosis in human neuroblastoma [34, 35]. The *lnc-LAMC2–1:1* polymorphism is located in the *LAMC1* gene and close to the *LAMC2* gene. Rs2147578 is in the first exon of *lnc-LAMC2–1:1*, and the 26th intron of the *LAMC1* gene near the *LAMC2* gene [21]. Carriers with the G allele have a slightly increased expression of *lnc-LAMC2–1:1* through binding between rs2147578G and *miR-128-3p* [21]. Previous evidence also suggests that the *lnc-LAMC2–1:1* rs2147578 C > G  polymorphism may contribute to childhood ALL development [22]. Our results show that the *lnc-LAMC2–1:1* rs2147578 C > G polymorphism may also be involved in neuroblastoma tumorigenesis.

Abnormal expression of *LAMC2* was found in several types of cancer, and elevated expression of *LAMC2* is associated with poor clinical outcome and relapse [36, 37]. *LAMC2* can interact with the epidermal growth factor receptor (*EGFR*), and influence its downstream pathway [38]. Previous studies revealed that the EGF receptor is overexpressed in neuroblastoma tissues and cells, and anti-EGFR agents are potential targeted therapies for neuroblastoma [39–41]. The possible interaction between the *lnc-LAMC2–1:1* rs2147578 polymorphism and the EGFR pathway may account for the increased risk of neuroblastoma of the G allele.

Our results from Guangdong (Southern China) and Henan (Northern China) provinces were inconsistent. In the Henan province subgroup, the CG genotype distribution was significantly higher in the neuroblastoma group, and subjects with the GG and CG genotypes had a significantly increased risk of neuroblastoma. In contrast, no association between the G allele and neuroblastoma was found in the Guangdong province subgroup. A possible explanation for this inconsistency may be the relatively complex genetic background of the Guangdong Chinese Han population. Studies on Y-chromosome phylogeny suggest that people in Southern China, including Guangdong province, are much more polymorphic than populations in Northern China, including Henan province [42–44]. However, the relatively small sample size of our study may introduce bias.

## Conclusion

The *Lnc-LAMC2–1:1* rs2147578 C > G polymorphism is associated with increased neuroblastoma susceptibility in Han populations of Northern China. Female individuals and children younger than 18 months with such genetic variants are at an increased risk for neuroblastoma. But with samples collected from only two provinces, we can’t make any solid conclusion. We might look into this question in the near future when we collect more samples.

## Additional file

Additional file 1: **Table S1.** Clinical characteristics of neuroblastoma cases and cancer-free controls. (DOCX 15 kb)

## Abbreviations

ALK: Anaplastic lymphoma kinase
ALL: Acute lymphoblastic leukemia
CI: Confidence interval
EGFR: Epidermal growth factor receptor
GWAS: Genome-wide association study
INSS: International Neuroblastoma Staging System
LncRNA: Long non-coding RNAs
OR: Odds ratio
SNP: Single nucleotide polymorphism
