# Supplementary material for: The rs2147578 C > G polymorphism in the Inc-LAMC2–1:1 gene is associated with increased neuroblastoma risk in the Henan children
Source: BMC Cancer. 2018 Oct 3;18:948. doi: 10.1186/s12885-018-4847-y (PMC6171233; doi:10.1186/s12885-018-4847-y)
Supplement: Supplementary file 1 — Table S1. Clinical characteristics of neuroblastoma cases and cancer-free controls. (DOCX 15 kb) [file 12885_2018_4847_MOESM1_ESM.docx]

| **Supplemental Table 1.** Clinical characteristics of neuroblastoma cases and cancer-free controls | | | | | | | | | | |
| --- | --- | --- | --- | --- | --- | --- | --- | --- | --- | --- |
| Variable | Guangdong province | | | | | Henan province | | | | |
|  | Case (n=275) | | Control (n=531) | | *P* ^a^ | Case (n=118) | | Control (n=281) | | *P* ^a^ |
|  | No. | % | No. | % |  | No. | % | No. | % |  |
| Age range, month | 0-132 | | 0.07-156 | | 0.229 | 0-131.1 | | 0.1-144.0 | | 0.484 |
| Mean ± SD | 31.50±25.43 | | 29.73±24.86 | |  | 46.24±29.98 | | 44.97±33.23 | |  |
| <12 | 70 | 25.45 | 145 | 27.31 |  | 9 | 7.63 | 32 | 11.39 |  |
| 12-60 | 177 | 64.36 | 313 | 58.95 |  | 76 | 64.41 | 179 | 63.70 |  |
| >60 | 28 | 10.18 | 73 | 13.75 |  | 33 | 27.97 | 70 | 24.91 |  |
| Gender |  |  |  |  | 0.510 |  |  |  |  | 0.196 |
| Female | 114 | 41.45 | 233 | 43.88 |  | 54 | 45.76 | 109 | 38.79 |  |
| Male | 161 | 58.55 | 298 | 56.12 |  | 64 | 54.24 | 172 | 61.21 |  |
| Clinical stage |  |  |  |  |  |  |  |  |  |  |
| 1 | 54 | 19.64 |  |  |  | 15 | 12.71 |  |  |  |
| 2 | 62 | 22.55 |  |  |  | 31 | 26.27 |  |  |  |
| 3 | 49 | 17.82 |  |  |  | 19 | 16.10 |  |  |  |
| 4 | 94 | 34.18 |  |  |  | 49 | 41.53 |  |  |  |
| 4s | 8 | 2.91 |  |  |  | 3 | 2.54 |  |  |  |
| NA | 8 | 2.91 |  |  |  | 1 | 0.85 |  |  |  |
| Site of origin |  |  |  |  |  |  |  |  |  |  |
| Adrenal gland | 64 | 23.27 |  |  |  | 89 | 75.42 |  |  |  |
| Retroperitoneal region | 87 | 31.64 |  |  |  | / | / |  |  |  |
| Mediastinum | 90 | 32.73 |  |  |  | 19 | 16.10 |  |  |  |
| Other region | 26 | 9.45 |  |  |  | 10 | 8.47 |  |  |  |
| NA | 8 | 2.91 |  |  |  | / | / |  |  |  |
| SD, standard deviation; NA, not available.  ^a^ Two-sided *χ^2^* test for distribution in neuroblastoma cases and cancer-free controls. | | | | | | | | | | |
